# Non-classical phenotypes of mismatch repair deficiency and microsatellite instability in primary and metastatic tumors at different sites in Lynch syndrome

**DOI:** 10.3389/fonc.2022.1004469

**Published:** 2022-12-15

**Authors:** Zhiyu Li, Bo Cheng, Shan Liu, Shanshan Ding, Jinhong Liu, Lanju Quan, Yanjiao Hao, Lin Xu, Huan Zhao, Jing Guo, Suozhu Sun

**Affiliations:** ^1^ School of Basic Medicine, Hebei North University, Zhangjiakou, Hebei, China; ^2^ Department of Pathology, Chinese People’s Liberation Army (PLA) Rocket Force Specialized Medical Center, Beijing, China

**Keywords:** microsatellite instability, colorectal cancer, Lynch syndrome, heterogeneity, mismatch repair protein

## Abstract

**Background:**

Lynch syndrome is a genetic disease characterized by abnormal DNA replication caused by germline variation in the mismatch repair (MMR) gene. There are rare non-classical phenotypes with loss of MMR protein expression and inconsistent microsatellite stability (MSS) in Lynch syndrome-related colorectal cancers. However, the difference between microsatellite instability (MSI) of extraintestinal tumors in a patient with Lynch syndrome has been closely studied. Herein, we reported the non-classical phenotypes of mismatch repair deficiency (dMMR) and MSI in four cases of Lynch syndrome in patients with colorectal cancer and other primary and metastatic tumors.

**Methods:**

A retrospective analysis was conducted on four patients diagnosed with Lynch syndrome between 2018 and 2022 in the Department of Pathology of the Rocket Forces Specialized Medical Center. A one-step immunohistochemical (IHC) assay was employed to detect loss in the expression of Lynch syndrome-associated MMR proteins (MLH1, PMS2, MSH2, and MSH6). MSI detection was performed in both primary and metastatic tumors at different sites in the four patients using NCI 2B3D (BAT25, BAT26, D2S123, D17S250, and D5S346) and single nucleotide site (BAT25, BAT26, NR21, NR24, NR27, and MONO27) methods. In addition, related MMR gene germline variation, somatic mutations, and *MLH1* gene promoter methylation were analyzed using next-generation sequencing and TaqMan probe-based methylation-specific polymerase chain reaction (MethyLight).

**Results:**

Two of the four patients were heterozygous for *MSH6* germline pathogenic variation, and the other two were heterozygous for *MSH2* germline pathogenic variation. In all cases, IHC detection of protein expression of the MMR gene with germline variation was negative in all primary and metastatic tumors; non-classical phenotypes of dMMR and MSI were present between primary and metastatic tumors at different sites. dMMR in Lynch colorectal cancer demonstrated high MSI, whereas MSI in primary and metastatic tumors outside the intestine mostly exhibited MSS or low MSI.

**Conclusions:**

The non-classical dMMR and MSI phenotype are mostly observed in Lynch syndrome, even in the context of MMR protein expression loss. Extraintestinal tumors infrequently present with a high degree of MSI and often exhibit a stable or low degree of MSI.

## 1 Introduction

Lynch syndrome is an abnormal DNA replication function disorder caused by germline variation in the mismatch repair (MMR) gene. In Lynch syndrome families, colorectal cancer, endometrioid adenocarcinoma, and other sites associated with MMR gene abnormalities are prone to early and frequent tumor development (1). Tumor tissues from patients with Lynch syndrome carry germline variation in the MMR gene and are generally associated with MMR protein expression deficiency (dMMR) and high microsatellite instability (MSI-H). Although rare, the heterogeneous expression of MMR at the protein level has been reported in several papers (2–4). This was manifested by the presence of clear MMR protein-positive expression areas within the regions of MMR expression loss in tumor tissues. They can be classified into four types: clonal, intraglandular, mixed, and isolated, according to the distribution characteristics of the lesions (5). Classical MMR-loss tumors usually present with MLH1/PMS2 or MSH2/MSH6 co-expression loss with MSI-H and the isolated expression loss of PMS2 or MSH6 with MSI-H,. Additionally, non-classical phenotypes of dMMR with MSI, though rare, have been reported in the literature, mainly in the form of compound loss of MMR protein, loss of canonical expression of MLH1/PMS2 or MSH2/MSH6 with microsatellite stability (MSS) or low microsatellite instability (MSI-L) (6), and intact expression of the four MMR proteins with MSI-H or MSI-L. The inconsistency between MMR protein expression and MSI in Lynch syndrome-associated tumors has occasionally been reported.

## 2 Materials and methods

### 2.1 Patients

Four cases of Lynch syndrome probands admitted to the Rocket Army Specialized Medical Center between 2018 and 2022 were selected. Paraffin-embedded tumor tissues from different sites were collected for MMR protein (MLH1, PMS2, MSH2, and MSH6) detection, MSI detection, MLH1 promoter methylation, next-generation sequencing (NGS), and tumor MMR germline and somatic gene mutation detection. All selected wax blocks were reviewed and confirmed by two senior pathologists, and informed consent was obtained from all selected cases. None of the patients had received radiotherapy, chemotherapy, immunotherapy, or neoadjuvant therapy before surgery.

### 2.2 Immunohistochemical detection of MMR proteins

A Roche Bench MarkXT automated immunohistochemistry instrument (Roche Biotechnology Development Co., Ltd., USA) was used for IHC analysis. The two-step EnVision method was used in this study. Primary antibodies (MLH1, PMS2, MSH2, and MSH6) and secondary antibodies were purchased from Roche Biotechnology Development Co. Ltd. Interpretation criteria were as follows: MLH1, PMS2, MSH2, and MSH6 positive signals were located in the nucleus; positively stained tumor and surrounding mesenchymal nuclei were brownish; negatively stained tumor cell nuclei were not stained, and the normal epithelium or mesenchymal tissue surrounding the tumor was brownish.

### 2.3 MLH1 promoter methylation assay

DNA was extracted from the tumor tissue. The DNA concentration and purity were determined using a UV spectrophotometer (PerkinElmer, USA). Sulfite modification and DNA extraction after modification were performed using the EZ DNA Methylation-GoldTM detection kit (ZYMO RESEARCH, USA) according to the manufacturer’s instructions. MLH1 methylation changes were detected using a 7500-fluorescence quantitative polymerase chain reaction (PCR) instrument (Thermo Fisher, USA). Simultaneously, COLO2A1 was used as an internal reference to calculate the relative expression of methylated genes. The primer and probe sequences were as follows: forward, CGTTATATATATCGTTCGTAGTATTCGTGTTT; reverse, CTATCGCCGCCTCATCGT; and probe, 6FAM-CGCGACGTCAAACGCCACTACG-TAMRA. The reaction system was: 5.2 μL of ddH_2_O, 2 μL of modified DNA, 10 μL of Premix Ex TaqTM Hot Start, 1.2 μL of upstream primer, 1.2 μL of downstream primer, and 0.4 μL of the probe to make a total volume of 20 μL. Tumor tissue DNA with a clear positive MLH1 promoter was used as a positive internal control, and triple distilled water was used as a negative internal control.

### 2.4 MMR gene germline variation detection in peripheral blood and MMR gene somatic mutation detection in tumor tissues

Peripheral blood and primary and metastatic tumor paraffin tissues were collected from the patients, and MMR germline and somatic mutations were detected by NGS whole-exome high-throughput sequencing. Additionally, the MSI status of tumor tissues was evaluated. The detection kits were purchased from Shanghai Kunyuan Gene Technology Co., Ltd. and Beijing Nuohezhiyuan Technology Co., Ltd. DNA extraction, library construction, on-machine sequencing, and data analysis were performed according to the manufacturer’s instructions.

### 2.5 MSI detection in primary and metastatic tumor tissues

NCI 2B3D (BAT25, BAT26, D2S123, D17S250, and D5S346) panels, based on fluorescence quantitative PCR-capillary electrophoresis, were used for primary and metastatic tumor tissue MSI detection. The 2B3D panel was established by the laboratory based on the nucleotide sites and primer sequences recommended by the NCI. Single-nucleotide site detection kits were provided by Microread Gene Technology Co. (Beijing, China). DNA was extracted from normal and tumor tissues and the DNA products were used as templates for PCR amplification. The 2B3D panel reaction products were analyzed by capillary electrophoresis using an ABI3500dx gene sequencer (Thermo Fisher, USA). Single nucleotide site amplification products were detected using a Microread Genetic Analyzer (Microread Genetics Co., Beijing, China). MSI-H was defined as two or more unstable detected sites; MSI-L was defined as one unstable detected site, and MSS was defined as all stable sites. The same experiment was repeated three times using the two methods. The results of the two methods, if consistent six times, were recorded as consistent experimental results, and if inconsistent once, were considered inconsistent experimental results.

## 3 Case history and results

### 3.1 Case 1

Case 1, a female patient, 80 years old, with intermittent blood in the stool for 2 years and worsening blood in the stool with increased frequency of stool for more than 2 months. Magnetic resonance imaging exhibited a cauliflower-like mass in the lower rectum with significant narrowing of the intestinal lumen. A large cystic pelvic mass with solid mural nodules was observed, which was closely related to the myometrium at the fundus of the uterus. A large cystic pelvic mass was adherent to the right pelvic wall, right adnexal area, and left adnexal area intraoperatively and was suspected to be of uterine fundus origin. Laparoscopic transabdominal perineal surgery combined with radical rectal cancer surgery, laparoscopic-assisted permanent sigmoid stoma, and laparoscopic pelvic mass resection were performed. A right lower abdominal mass was found 5 months after surgery, which was considered recurrent or metastatic. Therefore, surgical resection was performed.

**Figure 1:**
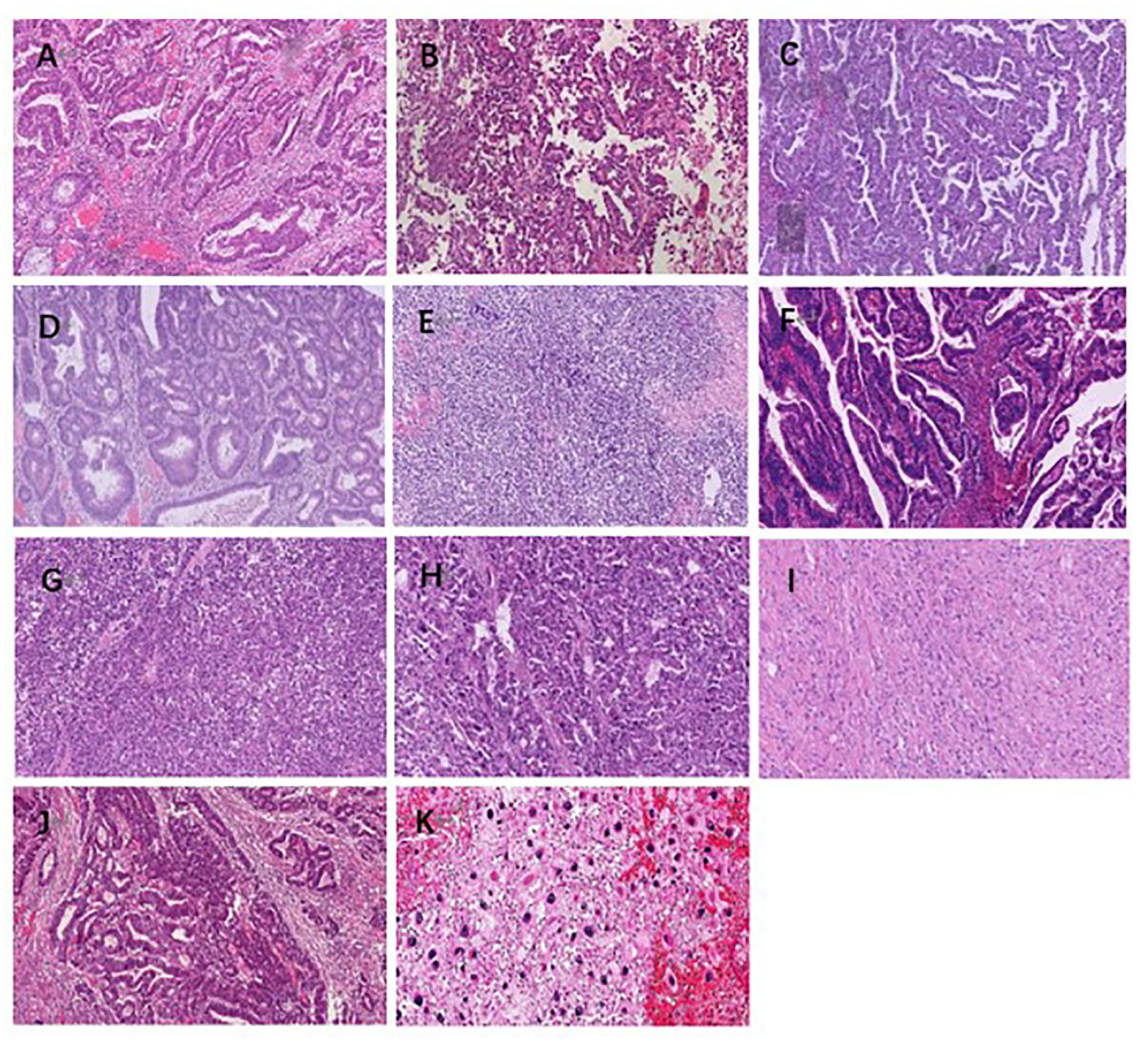
Case 1-4 HE staining diagram, 10x10. **(A)**: Case1 moderately differentiated adenocarcinoma of the rectum; **(B)**: Case1 uterine clear cell carcinoma in the pelvic region; **(C)**: Case1 clear cell carcinoma of the uterus metastasized to the right lower abdomen; **(D)**: Case2 well differentiated intramucosal carcinoma of the transverse colon; **(E)**: Case2 poorly differentiated carcinoma of bladder; **(F)**: Case3 moderately differentiated adenocarcinoma of the rectum; **(G)**: Case3 right adrenocortical carcinoma; **(H)**: Case3 intraperitoneal metastasis of adrenocortical adenocarcinoma; **(I)**: Case3 pleomorphic sarcoma on the right chest wall **(J)**: Case4 colon moderately differentiated adenocarcinoma; **(K)**: Case4 histiocytic sarcoma of the right upper arm.

**Figure 2:**
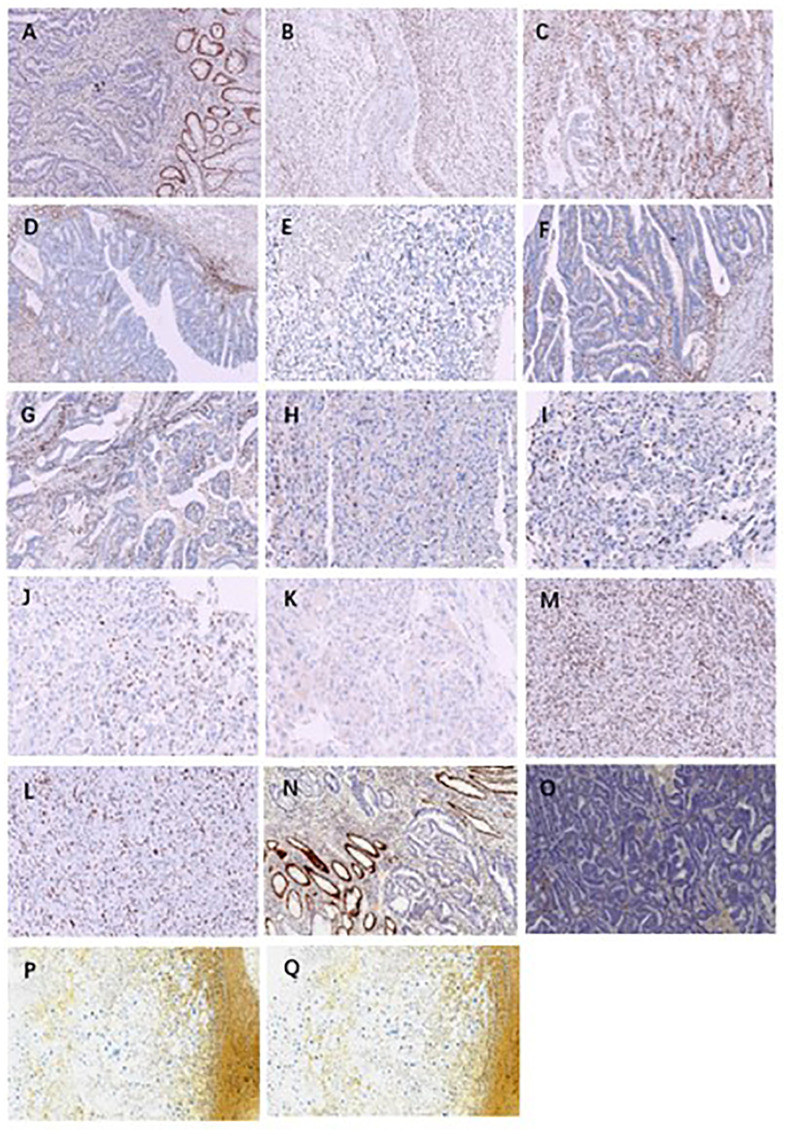
Immunohistochemical staining of cases 1-4. **(A)**: case 1 rectal moderately differentiated adenocarcinoma MSH6 expression loss; **(B)**: Case1 pelvic uterine clear cell carcinoma MSH6 expression loss; **(C)**: Case1 metastatic clear cell carcinoma of the uterus in the right lower abdomen MSH6 expression loss; **(D)**: Case2 well differentiated intramucosal carcinoma of transverse colon MSH6 expression loss; **(E)**: Case2 poorly differentiated carcinoma of bladder MSH6 expression loss; **(F)** and **(G)**: Case3 moderately differentiated adenocarcinoma of rectum MSH2 and MSH6 expression loss; **(H)** and **(I)**: Case3 adrenocortical carcinoma MSH2 and MSH6 expression loss; **(J)** and **(K)**: Case3 Abdominal metastatic adrenocortical carcinoma MSH2 and MSH6 expression loss; **(M)** and **(L)**: Case3 pleomorphic sarcoma of right chest wall MSH2 and MSH6 expression loss; **(N)** and **(O)**: Case4 moderately differentiated adenocarcinoma of colon MSH2 and MSH6 expression loss; **(P)** and **(Q)**: Case4 histiocytic sarcoma of the right upper arm MSH2 and MSH6 expression loss.

**Figure 3:**
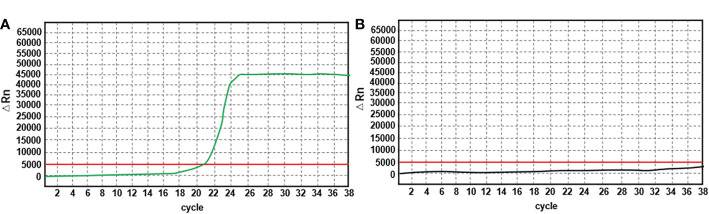
Reference graph of MLH1 methylation assay. **(A)**: Green line represents the fluorescence curve of the positive reference detection target; **(B)**: Black line is the fluorescence curve of the negative reference detection target; the Red line indicates the set threshold value. The horizontal coordinate represents the number of cycles and the vertical coordinate represents the signal value.

**Figure 4:**
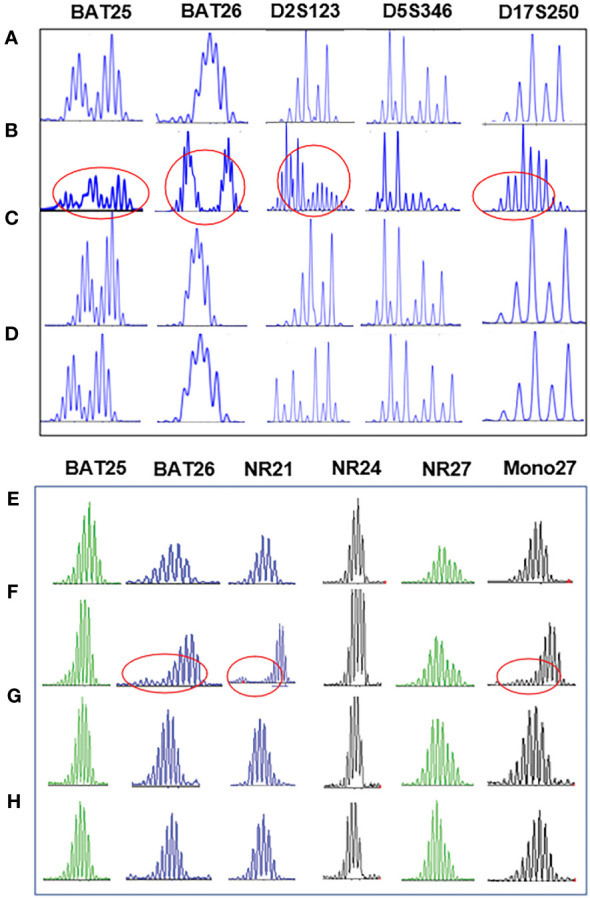
Detection results of NCI 2B3D and single nucleotide site panel of case1 based on fluorescence quantitative PCR capillary electrophoresis. Unstable sites are displayed at the mark. **(A)**: Normal control tissue; **(B)**: In rectal adenocarcinoma tumor tissue, 2B3D panel detection revealed that BAT25, BAT26, D2S123, and D17S250 sites were unstable, and the detection result was MSI-H; **(C)**: In pelvic endometrial clear cell carcinoma, 2B3D panel detection demonstrated that all sites were stable, and the detection result was MSS; **(D)**: In the metastatic tissue of endometrial clear cell carcinoma in the right lower abdomen, 2B3D panel detection suggested that all sites were stable, and the detection result was MSS; **(E)**: Normal control tissue; **(F)**: In rectal adenocarcinoma tumor tissue, single nucleotide site panel detection implied that BAT26, NR21, and Mono27 sites were unstable, and the detection result was MSI-H; **(G)**: In pelvic endometrial clear cell carcinoma, single nucleotide site panel detection unveiled that all sites were stable, and the detection result was MSS; **(H)**: In the metastatic tissue of endometrial clear cell carcinoma in the right lower abdomen, the panel detection of single nucleotide sites uncovered that all sites were stable, and the detection result was MSS.

The pathological investigation (**Table 1**) revealed that the rectal tumor was an invasive moderately differentiated tubular adenocarcinoma, with tumor cells invading the entire intestinal wall and a large number of lymphocytes infiltrating the mesenchyme, and mucinous adenocarcinoma structures could be seen locally (**Figure 1A**). The endometriotic cystic nodule attached to the wall was a clear cell carcinoma, with a microcystic papillary structure and clear or eosinophilic tumor cell cytoplasm, and Hobnail cells could be seen in the glandular lumen (**Figure 1B**). The metastatic mass in the right lower abdomen was also consistent with the morphological and IHC features of clear cell carcinoma; however, the cell grade and proliferation index were significantly higher than that of the primary lesion (**Figure 1C**). Upon immunohistochemistry, MMR proteins showed consistent loss of MSH6 protein expression in the rectal mass (**Figure 2A**), solid mural nodules of endometriotic cysts (**Figure 2B**), and right lower abdominal metastatic mass (**Figure 2C**), and positive PMS2, MSH2, and MLH1 expressions. The rectal mass, solid mural nodules, and right lower abdominal metastatic mass were negative in the MLH1 promoter methylation assay (**Figure 3**). Pathogenic variations in the germline of intron 7 of the *MSH6* gene (c.3646+1G>T) were detected in the peripheral blood, and the proband of Lynch syndrome was confirmed with family analysis and clinicopathological investigation (**Table 2**). Pathogenic variations in the *MSH6* gene system (c.676G>T) and suspected pathogenic variations in *MSH6* (c.3284G>A) were detected in the rectal mass; suspected pathogenic variations in the *MSH3* gene (c.190C>G) were detected in the pelvic mass, but no somatic mutations in the MMR gene were detected in the right lower abdominal mass (**Table 2**). MSI status of primary and metastatic tumor tissues was detected by NCI 2B3D and a single nucleotide locus panel, showing MSI-H in the rectal mass and MSS in both primary and metastatic clear cell carcinoma of the uterus and abdominal wall (**Figure 4**, **Table 3**).

**Table 1:** Clinicopathological features.

Case	Age	Gender	Site	Histology	Differentiation
Case1	80	F	Rectum	Tubular adenocarcinoma	Moderate
			Pelvic cavity	Clear cell carcinoma of the endometrium	Moderate
			Right lower abdominal mass	Metastasis of endometrial clear cell carcinoma	Moderate
Case2	62	M	Transverse colon	Intramucosal carcinoma	Well
			Bladder	Urothelial carcinoma	Poor
Case3	57	M	Rectum	Tubular adenocarcinoma	Moderate
			Adrenal gland	Cortical carcinoma	Well
			Abdominal cavity	Metastasis of cortical carcinoma	Moderate
			Right chest wall	Pleomorphic sarcoma	Poor
Case4	41	M	Colon	Tubular adenocarcinoma	Moderate
			Right upper arm	Histiocytic sarcoma	Poor

F, female; M, male; Site, Tumor site.

**Table 2:** Summary of germline variations and somatic mutations of mismatch repair genes.

**Case**	**Site**	**Germline mutation**	**Somatic Mutation**
Case1	Rectum	*MSH6*.c.3646+1G>T(50%)Pathogenic variation	*TP53*. c.844C>T(23%) Suspected pathogenic mutation *MSH6*. c.676G>T(20.34%) Suspected pathogenic mutation *MSH6*. c.3284G>A(23.44) Ambiguous mutation
	Pelvic cavity		*MSH3*. c.190C>G(27.37) Ambiguous mutation *CHEK2*. c.1117A>G (14.78%) Ambiguous mutation
	Right lower abdominal mass		*ATM.* c.8545C>T(28.29%) Suspected pathogenic mutation *ATM* .c.9022C>T (26.23%) Suspected pathogenic mutation
Case2	Transverse colon	*MSH6*.c.3752_3755dupCATT(42.27%) Pathogenic variation	*MSH6*. c.2105C>G (39.45%) Pathogenic mutation *TP53.* c.916C>T(33.21%) Pathogenic mutation
	Bladder		*TP53*. c.916C>T(33.3%) Ambiguous mutation *APC*. c.694C>T(4.5%) Ambiguous mutation
Case3	Rectum	*MSH2*. Exon copy number variation(1.12) Pathogenic variation *PMS2* .c.1883G>A(47.19%) Ambiguous variation	*MSH2.* c.1662-1G>A(37.45%) Pathogenic mutation *MSH2*. c.1963G>A(1.15%)Ambiguous mutation
	Adrenal gland		/
	Abdominal cavity		*MSH6*. c.3538T>G(25.07%)Ambiguous mutation
	Right chest wall		*MSH6*. c.3101G>A(1.52%) Ambiguous mutation
			*MLH1.* c.833C>T(1.14%) Ambiguous mutation
Case4	Colon	*MSH2*.c.1465G>T(50.92% ) Pathogenic variation	/
	Right upper arm		/

Percentage is the proportion of mutations detected; The value 1.12 indicates copy number variation, the value is close to 2 the result indicates negative, the value is close to 1 the result indicates positive, and the value is close to 4 the result indicates copy number increase; Pathogenic variants indicate that the variant affects protein expression, and suspected pathogenic variants indicate that the protein maybe affect protein expression.

**Table 3:** Summary of single nucleotide sites and NCI 2B3Dpanel test results.

Case	Site	Single nucleotide site Panel detection	2B3D Panel detection
Case1	Rectum	MSI-H	MSI-H
	Pelvic cavity	MSS	MSS
	Right lower abdominal mass	MSS	MSS
Case2	Transverse colon	MSI-H	MSI-H
	Bladder	MSI-L	MSI-L
Case3	Rectum	MSI-H	MSI-H
	Adrenal gland	MSS	MSS
	Abdominal cavity	MSI-H	MSI-H
	Right chest wall	MSI-L	MSI-L
Case4	Colon	MSI-H	MSI-H
	Right upper arm	MSS	MSS

MSI-H, microsatellite high instability; MSI-L, microsatellite low instability; MSS, microsatellite stability.

### 3.2 Case 2

A 62-year-old man was admitted to our hospital with intermittent gross painless hematuria for more than 1 month. CTU of the urinary tract revealed an occupying lesion on the left wall of the bladder, left kidney, and left ureter. Colonoscopy revealed a bulging mass in the transverse colon, approximately 43 cm from the anal verge, occupying approximately 1/3 of the intestinal lumen. Laparoscopic radical cystectomy, bilateral ureteral skin fistulas, lymph node dissection, and laparoscopic radical colon cancer surgery were performed.

Pathological investigation (**Table 1**) revealed a high-grade adenomatous polyp with a localized carcinoma (intramucosal carcinoma) in the transverse colon (**Figure 1D**). The bladder mass was a poorly differentiated invasive uroepithelial carcinoma with a "medullary carcinoma" structure, and a large interstitial infiltrate of lymphocyte-dominated inflammatory cells (**Figure 1E**). Upon immunohistochemistry, MMR proteins showed a consistent loss of MSH6 protein expression in the transverse colon mass (**Figure 2D**) and bladder mass (**Figure 2E**), with positive PMS2, MSH2, and MLH1 expressions. The transverse colon and bladder masses were negative for MLH1 promoter methylation testing. Germline pathogenic variations in exon 8 of *MSH6* (c.3752_3755dupCATT) were detected in the peripheral blood, and the proband of Lynch syndrome was confirmed with family analysis and clinicopathological investigation (**Table 2**). Pathogenic mutations in the *MSH6* gene system were detected in the transverse colon mass (c.2105C>G), whereas no somatic mutations in the MMR gene were detected in the bladder mass (**Table 2**). Different MSI panel assays showed MSI-H in the transverse colon mass and MSI-L in poorly differentiated bladder uroepithelial carcinomas (**Table 3**).

### 3.3 Case 3

A 57-year-old man was admitted to the hospital with an enlarged right abdominal mass that had persisted for more than 2 months. CT showed a large right intra-abdominal mass with a poorly demarcated liver, right diaphragm elevation, and left mediastinal shift. Additionally, multiple blood-rich lesions were observed in the right perirenal fat capsule, prehepatic fat space, and retroperitoneum. He underwent abdominal exploration, abdominal mass resection, intestinal adhesion release, and tumor resection in the right adrenal region. The patient had also undergone resection of a right chest wall mass 4 years earlier, followed by laparoscopic resection of an occupying adrenal area lesion 2 months later, and radical rectal cancer and pelvic mass 2 years ago.

Pathological investigation (**Table 1**) revealed that the rectal mass was an invasive moderately differentiated tubular adenocarcinoma with invasion of the muscularis propria and massive lymphocytic infiltration of the tumor mesenchyme (**Figure 1F**), which was an adrenal cortical adenocarcinoma with primary (**Figure 1G**) and metastatic tumor foci (**Figure 1H**). The tumor in the proximal adrenal region was highly differentiated, the distant metastatic foci were poorly differentiated, and the soft tissue tumor of the chest wall was undifferentiated pleomorphic sarcoma with spindle-shaped and polygonal tumor cells that invaded the striated muscle of the chest wall (**Figure 1I**). Upon immunohistochemistry, MMR proteins showed consistent loss of MSH2 and MSH6 protein expression in the rectal mass (**Figures 2F, G**), adrenal primary (**Figures 2H, I**), metastatic mass (**Figures 2J, K**), and right chest wall (**Figures 2M, L**) mass, with no heterogeneous expression of MMR protein, and positive expression of MLH1 and PMS2. The rectal mass, primary and metastatic adrenal masses, and right chest wall masses were negative for MLH1 promoter methylation. Pathogenic variations in exon 8 of the *MSH2* gene were detected in the peripheral blood (copy number variant with copy number deletion of 1.12), and the proband of Lynch syndrome was confirmed with family analysis and clinicopathological investigation (**Table 2**). Pathogenic systemic mutations in the *MSH2* gene (c.1662-1G>A) and suspected pathogenic mutations in the *MSH2* gene (c.1963G>A) were detected in the rectal mass, suspected pathogenic mutations in the *MSH6* gene (c.3538T>G) were detected in the adrenal metastatic tumor, *MSH6* gene (c.3101G>A) and *MLH1* (c.833C>T) with suspected pathogenic mutations, and no somatic mutations in the MMR gene were detected in the adrenal primary mass (**Table 2**). Different MSI panel assays showed MSI-H for rectal mass, MSI-L for undifferentiated pleomorphic sarcoma of the abdominal wall, MSS for primary adrenocortical carcinoma, and MSI-H for metastatic adrenal carcinoma (**Table 3**).

### 3.4 Case 4

A 41-year-old man was admitted to the hospital 4 months after a mass was found in his right upper arm. Ultrasound demonstrated a subcutaneous soft tissue hypoechoic mass of approximately 12.6×10.3×8.5 cm on the lateral right upper arm, which was poorly demarcated from surrounding tissues. The patient had undergone radical colon cancer treatment 4 months previously. The postoperative pathological diagnosis was a moderately differentiated tubular adenocarcinoma of the colon.

Pathological investigation (**Table 1**) revealed that the colon mass was a moderately to poorly differentiated invasive tubular adenocarcinoma with invasion of the serosal layer and massive lymphocytic infiltration of the tumor mesenchyme, with locally visible mucinous adenocarcinoma structures (**Figure 1J**). The right upper arm mass was a histiocytic sarcoma with round, polygonal tumor cells with abundant cytoplasm, large, deep-stained nuclei, obvious nucleoli, and scattered multinucleated tumor giant cells (**Figure 1K**). Upon immunohistochemistry, MMR proteins showed consistent loss of MSH2 and MSH6 protein expression in the colon mass (**Figures 2N, O**) and right upper arm mass (**Figures 2P, Q**), with no heterogeneous expression of MMR protein and positive expression of MLH1 and PMS2. The colon and right upper arm masses were negative for MLH1 promoter methylation. Pathogenic mutations (nonsense variations, c.1465 G>T, p.Glu489Ter) in exon 9 of *MSH2* were detected in the peripheral blood (**Table 2**). Somatic mutations in the MMR gene were not detected in the colonic or right upper arm mass (**Table 2**). Different MSI panel assays showed MSI-H in the colon mass and MSS in the right upper-arm histiocytic sarcoma (**Table 3**).

No methodological differences in the MSI assay results were observed in any of the cases. There were inconsistencies in MSS between colorectal cancer and extraintestinal tumors in the same patient with germline variations of the MMR gene and loss of MMR protein expression. MSI status may be consistent or inconsistent between extraintestinal primary and metastatic tumors. The consistency between germline variations of MMR genes and loss of MMR protein expression in primary and metastatic tumors outside the intestine did not primarily induce a high degree of MSI in this tumor.

## 4 Discussion

The Lynch syndrome is an autosomal dominant disorder caused by germline variations in the *MMR* gene; the incidence of colorectal cancer, endometrioid adenocarcinoma, and other tumors associated with *MMR* gene abnormalities is significantly higher in members of the patient's family. IHC analyses for MSH1,PMS2, MSH2, and MSH6 and MSI analysis of the tumor cells are important components of Lynch syndrome screening. Both methods have a high concordance (90%–97.5%) in Lynch-associated colorectal cancer (7), often revealing the classical MLH1/PMS2, MSH2/MSH6, PMS2, and MSH6 expression deficiency (dMMR) with MSI-H; however, both have a lower concordance in extraintestinal tumors, mostly showing dMMR with an MSI-L/MSS non-classical phenotype (6, 8, 9). Herein, we have reported four cases of the Lynch syndrome proband patients with simultaneous or heterozygous colorectal cancer and extraintestinal tumors (one each undergoing *MMR* germline and systemic mutation detection, IHC staining for MMR protein expression, or MSI analysis) to reveal the molecular characteristics and correlations of *MMR* gene mutation, MMR protein expression, and MSI among different tumors in the same patient.

In case 1 we observed the patient was diagnosed as the proband of the Lynch family at the age of 80 years; this is significantly older than the age generally observed in cases of the classical Lynch syndrome (≤50 years). Lynch syndrome caused by germline pathogenic variations in *MSH6* can have a late onset and is often accompanied by MMS or MSI-L (10). Because *MSH6* variations and loss of protein function mainly cause instability of single-nucleotide microsatellite repeat sequences (accumulation of DNA replication errors), You et al. attributed the dMMR/MSS dMMR/MSI-L (MSI non-classical phenotype caused by *MSH6* variations) to the presence of double nucleotide sites in the 2B3D (two single-nucleotide sites and three double-nucleotide sites) MSI assay panel caused by false negatives (10). To eliminate possible experimental errors due to experimental methods and tumor heterogeneity, we repeatedly validated different paraffin tissues from the same tumor using NCI 2B3D-based and single nucleotide locus panels, respectively, and no differences in the results that were clearly caused by the experimental methods and assay batches were observed. Germline variations in *MSH6* (chr2:48032847. c.3646+1G>T (50%)) were detected in the patient's rectal and uterine primary and abdominal metastatic masses. Furthermore, somatic mutations in *MSH6* (c.3284G>A, c.676G>T) were also detected in the rectal tumors; however, similar somatic mutations were not detected in the extraintestinal tumors, suggesting that differences in the frequency and type of secondary mutation strikes suffered by the *MMR* gene among different organs may be a potential factor that triggers the atypical phenotype of dMMR and MSI (11, 12).

In case 2 we observed the patient had a less-differentiated invasive uroepithelial carcinoma with medullary carcinoma features. The patient experienced painless hematuria as the first symptom, and clinical examination revealed a bladder mass. The mass showed a "medullary carcinoma" structure, and a large amount of lymphocyte-based inflammatory cell infiltration was observed in the interstitium; thus, a Lynch syndrome-related tumor was suspected. The diagnosis of the Lynch syndrome was confirmed by IHC staining for the MMR protein, MSI analysis, and germline variation detection of genetically related genes. Further, colonoscopy revealed a colonic mass, and pathological examination revealed a high-grade adenomatous polyp with localized carcinoma. In clinical practice, due to the lack of a clear family history and a history of bowel cancer, Lynch-associated extraintestinal tumors are often diagnosed as conventional sporadic tumors; thus, the opportunity to confirm the diagnosis of the Lynch syndrome is missed. Routine inclusion of MMR protein detection in tumor IHC staining can provide important clues for Lynch screening. An upper uroepithelial carcinoma with an *MSH2* germline variant is the most frequent, while bladder cancer is relatively rare, among Lynch-associated uroepithelial tumors (13, 14). Skeldon et al. found that patients with Lynch syndrome-associated *MMR* gene variations are not only prone to uroepithelial cancer, but also have a significantly higher risk of bladder cancer; thus, screening for Lynch-associated tumors should also include a screening for bladder cancer (15). Ekmekci et al. investigated the relationship between histopathological features and MSI in young patients (under 40 years of age) with uroepithelial carcinomas of the bladder and found that the tumor grade, tumor stage, degree of tumor differentiation, and tumor infiltrative growth pattern had a significant impact on the prognosis; however, they observed that MSI had no effective role in bladder carcinogenesis in these patients (16). The bladder tumor reported in case 2 was a hypodifferentiated uroepithelial carcinoma with MSH6-expression deficiency and MSI-L. The tumor cells were negative for CK5/6, P63, and P40 and positive for P53 and GATA3; thus, this tumor was a rare type of a uroepithelial carcinoma, but its features were consistent with the typical histological features of Lynch-related tumors.

We observed case 3 presented with an isolated, soft tissue, pleomorphic, undifferentiated sarcoma of the chest wall as the first symptom; an occupying lesion was detected in the right adrenal gland 2 months postoperatively following a pathological examination of a highly differentiated adrenocortical adenocarcinoma. Four years postoperatively, another giant abdominal occupancy developed, and the pathology was diagnosed as a recurrent, metastatic, moderate-to-poorly differentiated adrenocortical adenocarcinoma. As both soft tissue sarcomas and adrenal carcinomas are relatively rare Lynch-related tumors, IHC staining for the MMR protein and MSI analysis were not performed in the first two routine pathological examinations. In the third postoperative pathological examination, an IHC staining for the MMR protein was performed, and MSH2/MSH6 expression was found to be absent in the adrenal carcinoma tissue. The tortuous diagnosis of this patient demonstrates the positive role of IHC testing for the MMR protein in the detection of diagnostic clues for the Lynch syndrome. Lynch-associated adrenocortical adenocarcinomas are relatively rare, with an incidence of 3.2% in patients with ACC; this is comparable to the LS-associated prevalence of colorectal and endometrial cancers (17). Lynch-associated ACC is most commonly associated with *MSH2* germline variations, and its MMR protein IHC results are consistent with the germline variation status of the gene, commonly MSS (18). This is consistent with the test results in this case.

In both cases 3 and 4 we observed the patients presented with an extraintestinal, primary soft tissue sarcoma. The sarcoma in case 3 was an undifferentiated pleomorphic sarcoma of the chest wall, while the sarcoma in case 4 was a histiocytic sarcoma of the right upper arm. Sarcomas are rare mesenchymal tumors with marked heterogeneity, accounting for approximately 1% of all malignant tumors in adults. Sarcomas have been reported in patients with LS; these are mainly LS-associated sarcomas with MMR gene variation-carrying tumor tissue and protein-expression deficiencies and Lynch-associated sarcomas without these changes (19). Lynch-associated soft tissue sarcomas are mostly characterized by *MSH2* germline variations; in some cases, patients with these tumors exhibit the Muir–Torre syndrome with multiple cutaneous tumors and an atypical microsatellite phenotype (20). In our study, all cases were of LS-associated sarcomas and presented with *MSH2* germline variations and MSH2/MSH6 protein-expression deficiency; these features are similar to the molecular features of similar tumors reported in the literature (21).

The non-classical phenotype dMMR is closely related to the heterogeneous expression of the MMR protein; the latter is mainly manifested by the simultaneous presence of well-defined MMR protein-positive expression areas within regions of tumor tissue with MMR protein expression deficiency. According to the distribution characteristics, heterogeneous lesions can be classified into four subtypes: clonal, intraglandular, isolated, and mixed. These lesions differ from the lesions that stain unevenly during IHC staining due to issues with staining techniques (such as tissue fixation and preprocessing); they have specific molecular pathological mechanisms and significance. Joost et al. confirmed the existence of heterogeneous MMR-protein expression (as described above) in 14 colorectal cancers; variant expression of antigenic determinant clusters, antigenic expression associated with different differentiation states, secondary mutational strikes occurring in specific tumor clones, and alterations in the tumor microenvironment (such as methylation, hypoxia, and oxidative stress) can lead to the heterogeneous expression of MMR proteins (5). In our study, clear germline variations in the MMR gene were observed in the four cases, and no methylation of the promoter region of the *MLH1* gene was noted. Although secondary mutations (somatic mutations) in the MMR gene existed at different loci among the intestinal, extraintestinal, primary, and metastatic tumors in the same patient, they did not cause a heterogeneous MMR-protein expression; differences were mainly noted in the microsatellite status among tumors at different sites in the intestinal and extraintestinal regions within the same Lynch patients.

Inconsistency between the MMR and MSI phenotypes in the tumor tissue from the same site is rare in colorectal cancer but more common in extraintestinal LS-associated tumors. This may be due to false negatives caused by tumor-tissue heterogeneity or a misinterpretation of the IHC staining results for the MMR protein and MSI analysis results. The inconsistency between MMR and MSI phenotypes persisted even after the elimination of experimental errors. Jaffrelot et al. detected 585 cases of dMMR in 4,948 MMR test samples; 89 (15%) of these had non-classical phenotypes (mostly in genetic syndromes and extracolorectal tumors) (6). Shimozaki et al. reported that the non-classical phenotype of dMMR/MSS was also found in pancreatic cancer in patients with the Lynch syndrome (8); Latham et al. identified 37 Lynch syndrome-associated tumors with MSS from among 15,045 cases (with 50 types of tumors) (9); The MSI status of tumor tissue from the same tissue section of the same wax block with an heterogeneous MMR expression region can be clearly heterogeneous. McCarthy et al. used laser microdissection and NGS techniques to analyze the challenges to the interpretation of IHC results and MSI assessment posed by heterogeneous MMR-protein expression; they found that in one case of a colon adenocarcinoma with heterogeneously expressed MLH1 and PMS2, the MLH1/PMS2 expression-preserved region had MSS and the MLH1/PMS2 expression-lost region had high MSI. Furthermore, they identified one MSH6-positive gastric cancer infiltrating lesion with a complete loss of the MSH6 expression in its heterogeneous hyperplastic lesion; however, both regions were MSI-H and had the same MSH6 variant:c.3261delC (2). The above results confirm that the heterogeneous phenotypes of MMR and MSI in the same tumor tissue may have complex regulatory mechanisms. On the one hand, although the loss of function of The MMR protein caused by molecular events (such as mutation and methylation of the tumor MMR gene) can lead to tumor cells in the MSI-H state, the loss of function of the MMR protein can still be detected by IHC due to the fixed cluster of partial antigenic decisions. On the other hand, tumor subclones with different differentiation states have their own uniqueness at morphological, genetic, epigenetic, or transcriptomic levels, and show heterogeneous expression of MMR proteins with consistent functional and immunophenotypic associations and corresponding MSI states. In addition, there is functional compensation between MMR proteins (PMS2/PMS1, MSH6/MSH3), which can maintain the tumor MSS status in the absence of the four most frequently detected MMR proteins. All atypical dMMR phenotypes reported herein occurred in the extraintestinal tumor tissues, including two cases of soft tissue sarcomas due to germline variations in the *MSH2* gene, MSL-L, and MSS. A dMMR inconsistency with MSI was not seen in all primary colorectal cancers; this trend was consistent with the findings of Jaffrelot et al (6).

In this study, we found that the MSI status of LS-associated tumors differed significantly in the same patients with LS with the same MMR germline variation and MMR-protein expression deletion background; colorectal cancers showed more dMMR/MSI-H and extraintestinal tumors showed more dMMR/MSI-L and dMMR/MSS. The molecular mechanism of this non-classical phenotype has not been fully elucidated, but may involve a correlation of MSI with the systemic organ and tissue type. In one study, there were significant organ differences in the proportion MMR protein-expression deficiency coinciding with MSI-H; the proportions were as follows: 94%, colorectal cancer; 71%–87%, endometrioid carcinoma; and 38%–40%, epithelial carcinoma of the upper urinary tract (22). Kuismanen et al. found that the MSI profiles of colorectal and endometrial cancers differed significantly in patients with LS. In colorectal cancers, the dominant MSI sites contained both coding and non-coding mononucleoside sites; in endometrial cancers, a greater heterogeneity in the MSI sites was noted. Compared with colorectal cancer, endometrial cancer has a lower proportion of MSI loci and a shorter number of allelic shifts at single-nucleotide microsatellite loci (23). In our study the microsatellite loci characteristics were similar between the LS-associated extraintestinal tumors and endometrial cancer. The MSI analysis panel used in this study, based on the NCI 2B3D and Promega five single-nucleoside loci design, was approved by the National Pharmaceutical Agency and is suitable for tumor MSI detection in the Chinese population. Although these MSI assays were only used for MSI analysis of LS-related colorectal cancer in the early stage, they have now become the mainstream assays for pan-tumor MSI analyses. The results of these method in extraintestinal tumors were highly consistent with those of NGS of multi-MSI sites, indicating that the apparent differences in the MSI-H incidence among different tissues and organs in the context of germline variations of the same *MMR* gene and MMR protein-expression deletion are objective and not false negatives caused by assay defects (24).

Knudson observed that in hereditary retinoblastoma, germline variations alone are not sufficient to cause tumorigenesis; thus, they inferred that hereditary retinoblastoma involves two types of mutations (a germline variation and a somatic mutation), and proposed the "two-hit doctrine." This doctrine suggests that the second hit may be in the germline variation (one hit) which may be an intragenic mutation, whole-gene deletion, nondisjunction, or somatic recombination of the second allele of the same gene; this would eventually lead to somatic mutation or deletion of the normal allele and affect the cellular MMR system (25). dMMR crypt lesions were found around MMR protein-deficient adenomas by Ahadova et al. (11). The normal histological morphology of these lesions suggests that dMMR plays an important role in tumorigenesis. Lynch syndrome-associated CRC is also a sporadic adenomatous polyp in its early stages, and subsequent double-allele mutations and abnormal expression and function of the MMR protein increase the instability and mutational load of tumor-associated gene microsatellites; these then manifest as MSI-H and/or TMB-H, which in turn accelerate tumor development. This suggests that MSI lags behind the development of dMMR. The inconsistency between MMR protein and MSI can be detected when dMMR occurs, and the amount of tandem repeat sequence mutations in tumor cells does not accumulate to MSI (11, 12). In the Lynch syndrome, due to heterogeneity of the different organ tumors themselves, when germline variations occur in the MMR gene, there is also an inconsistency in the subsequent somatic mutations in the tumor cells of different tissues and organs. This difference in the frequency, locus, and type of mutations resulting from the superposition of germline and somatic mutations may also be an important reason for the inconsistent MSI status between primary and metastatic tumors of different organs in the same patient. In case 1, a germline *MSH6* variation [chr2:48032847. c.3646+1G>T (50%)] was detected in the rectal and uterine primary tumors and lower abdominal metastatic tumors. Furthermore, other somatic mutations of *MSH6* (c.3284G>A, c.676G>T) were detected in this patient's rectal tumors, suggesting that the frequency and type of secondary mutation strikes may differ among organs, resulting in the atypical phenotype of dMMR and MSI.

The mutation rate of microsatellites varies widely among motifs; it is significantly impacted by intrinsic DNA characteristics (gene motif size and sequence as well as the length of gene bundles). This variation is due to differences in the DNA polymerase error rates and in the correction efficiency of MMR gene in repetitive sequences of different sizes and sequences. Thus, the instability of single, dinucleotide, and tetranucleotide markers is generated by different signaling pathways, and multiple genomic maintenance pathways are involved in maintaining MSS (26). Although the same patients with intestinal, extraintestinal, primary, and metastatic tumors carry the same MMR germline variations and show no significant differences in the MMR protein-expression levels on IHC staining, different somatic secondary mutation sites and types of the MMR gene, polymorphic variants in the gene structure of tissue cells from different organs, and the immune microenvironment may have an impact on the microsatellite status of these cells, thus occurring in the same Lynch patient. The inconsistency in the state of tumor microsatellites among different lesion sites in the same Lynch patient. The complex molecular mechanism underlying this process is unclear and deserves a further in-depth study.

In this paper, we have reported four cases of non-classical phenotypes of MSI occurring in primary and metastatic intestinal and extraintestinal tumors in patients with the Lynch syndrome. Unlike the usual heterogeneous expression of the MMR protein occurring within the tumor tissues, these cases stand out for the inconsistency between the absence of an IHC expression of the MMR protein and the microsatellite status of tumor cells, a phenomenon that occurs in the same patient and shows a clear tendency to prefer extraintestinal tumors. Because extraintestinal tumors in Lynch syndrome are not always completely synchronized with colorectal cancer in terms of the time of occurrence, many patients present and operate with isolated extraintestinal tumors only, and it is difficult to confirm the diagnosis by histomorphological analysis alone if the history is incomplete. In the abovementioned four cases, MMR expression was found to be absent during routine MMR-protein testing, and the diagnosis was confirmed by tracing the family history of the tumor and performing additional MSI, MLH1 promoter methylation, and peripheral blood MMR germline variation testing. In addition, because of the low concordance between dMMR and MSI-H in many extraintestinal tumors, when we use the MSI assay as a routine concomitant diagnostic test for pan-tumor immunotherapy, we should refer to both MMR and PDL1IHC assessment and TMB assay results to avoid missing the treatment timing due to the presence of an MSS state in the absence of MMR protein expression.

## Data availability statement

The data presented in the study are deposited in the NCBI repository, accession number PRJNA868287.

## Ethics statement

The studies involving human participants were reviewed and approved by Ethics Committee of the Specialized Medical Center of the Rocket Forces. Written informed consent for participation was not required for this study in accordance with the national legislation and the institutional requirements.

## Author contributions

Conceptualization: ZL, SS. methodology: ZL, SS. software: ZL. validation: SS. formal analysis: ZL, LX, HZ. investigation: SL, SD, JL, LQ. resources: ZL, SS. data curation: ZL. writing (original draft preparation): ZL. writing (review and editing): ZL, SS, BC. visualization: ZL, YH. supervision: SS, BC. All authors contributed to the article and approved the submitted version.
