# Supplementary figures and images for: Non-classical phenotypes of mismatch repair deficiency and microsatellite instability in primary and metastatic tumors at different sites in Lynch syndrome
Source: Front Oncol. 2022 Dec 15;12:1004469. doi: 10.3389/fonc.2022.1004469 (PMC9797996; doi:10.3389/fonc.2022.1004469)

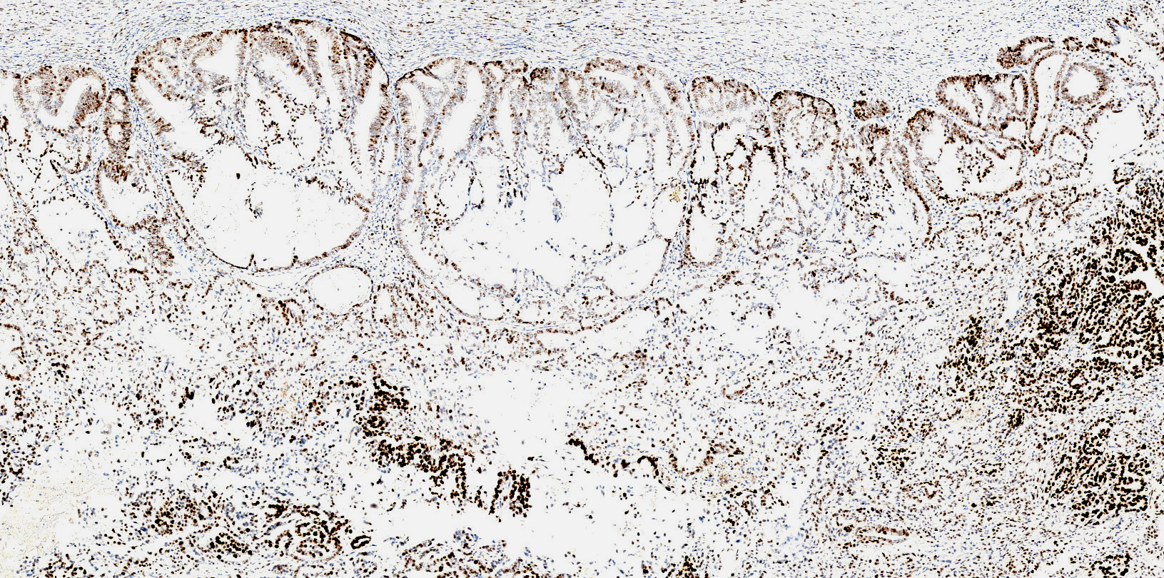

Supplement: Supplementary file 1 [file DataSheet_1.zip › Case 1/Pelvic cavity MLH1.png]

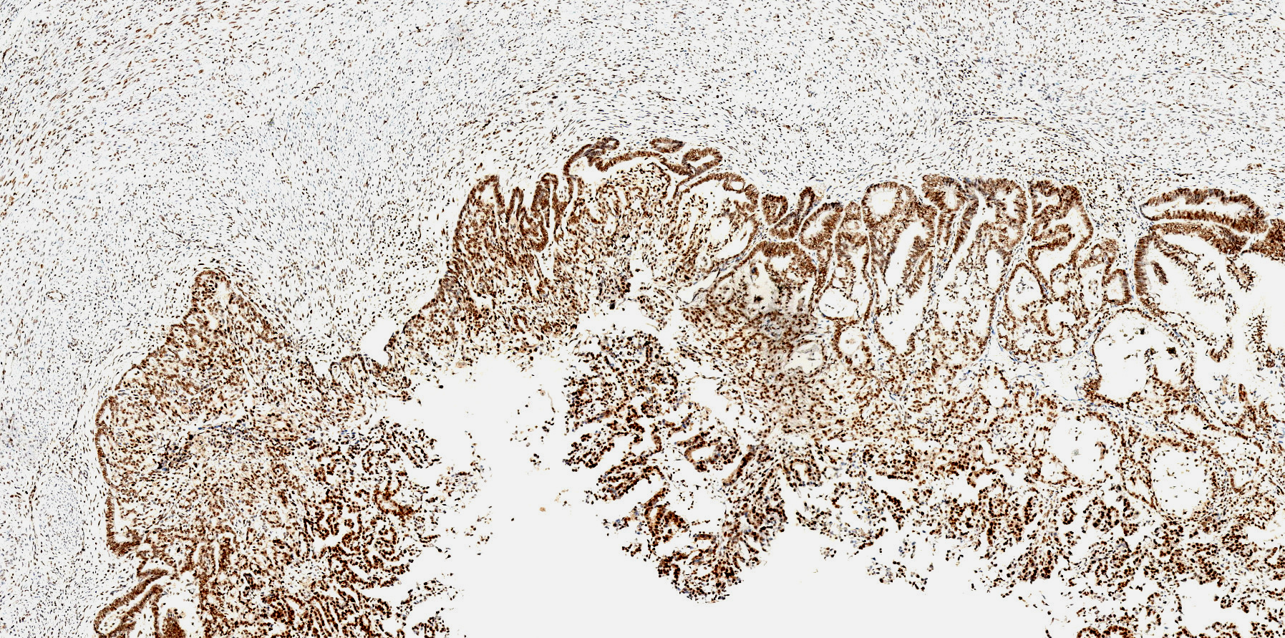

Supplement: Supplementary file 1 [file DataSheet_1.zip › Case 1/Pelvic cavity MSH2.png]

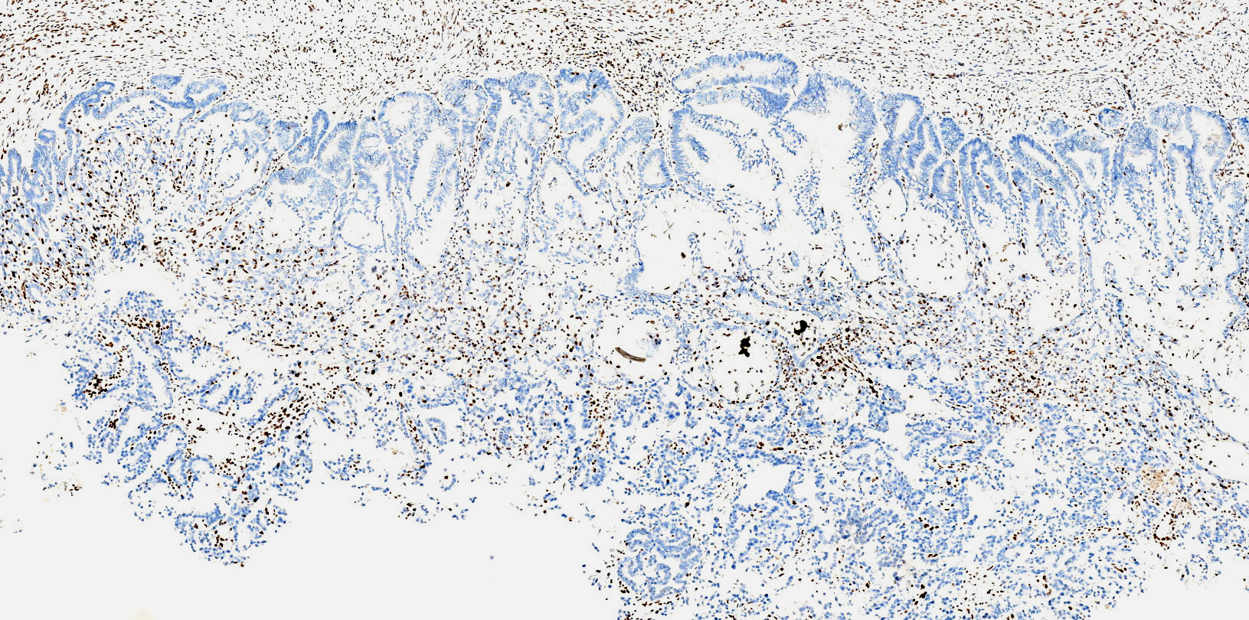

Supplement: Supplementary file 1 [file DataSheet_1.zip › Case 1/Pelvic cavity MSH6.png]

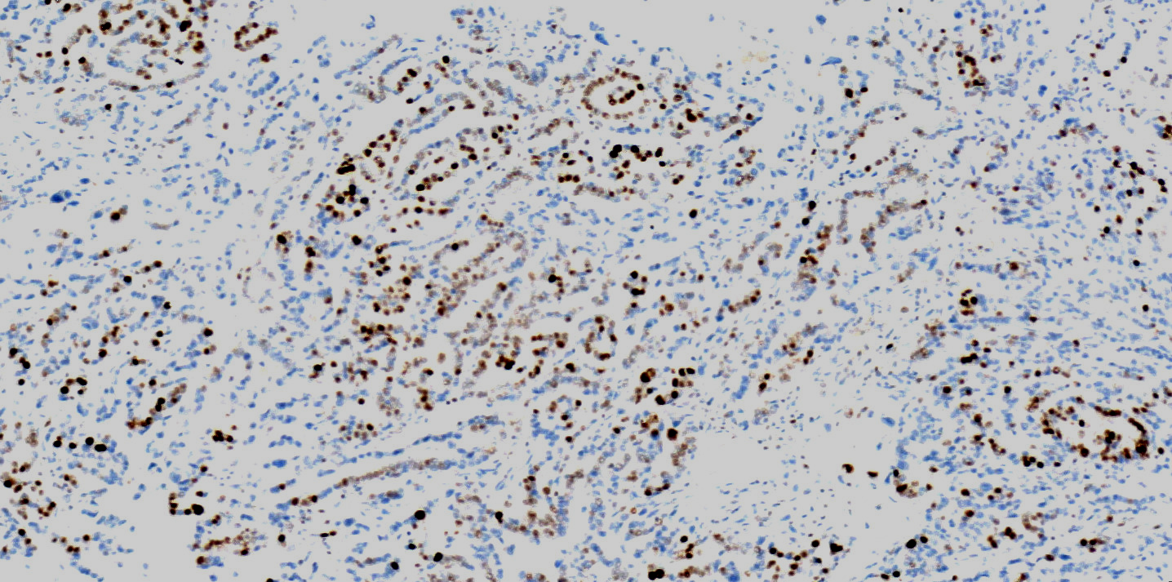

Supplement: Supplementary file 1 [file DataSheet_1.zip › Case 1/Pelvic cavity PMS2.png]

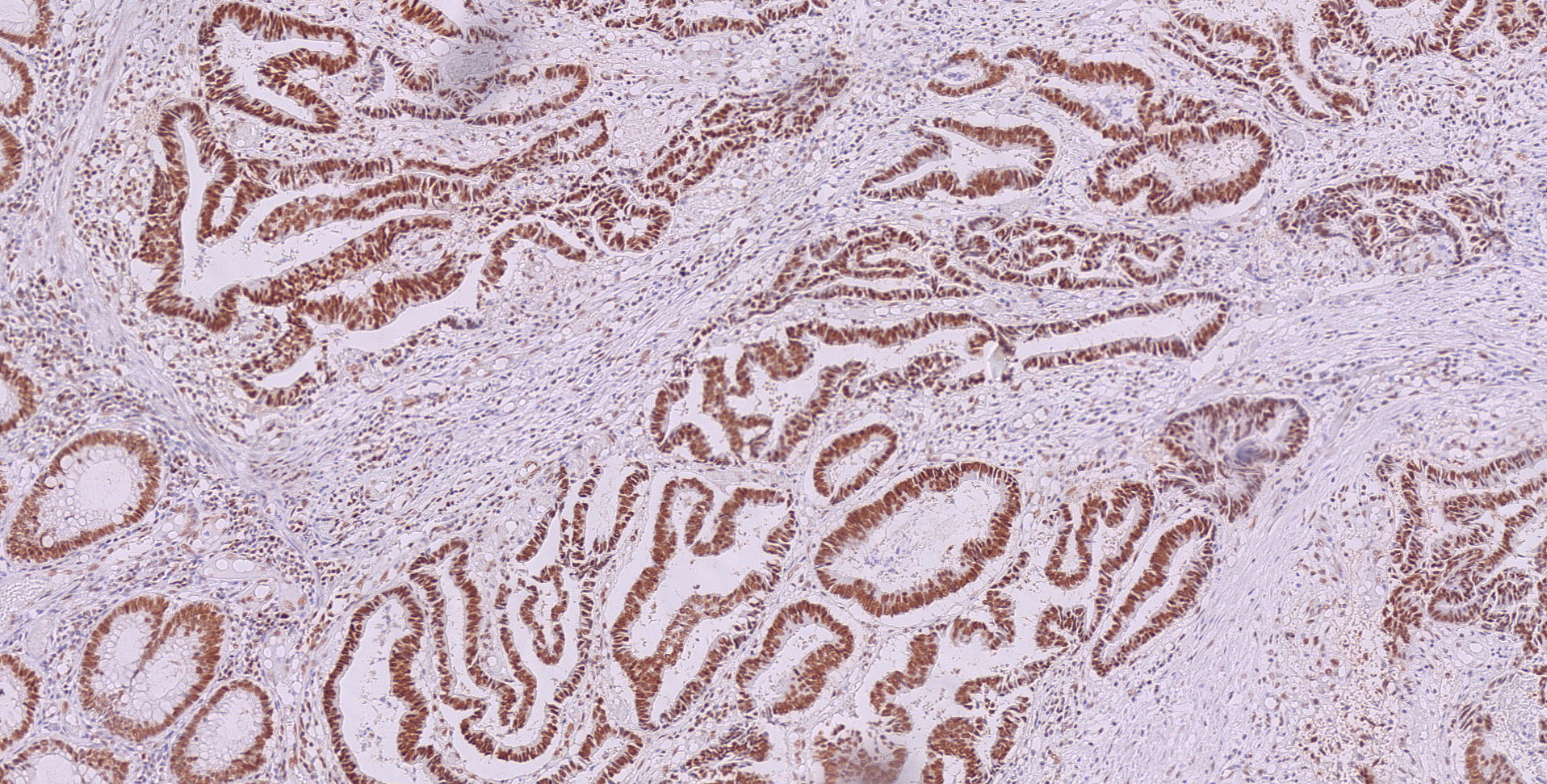

Supplement: Supplementary file 1 [file DataSheet_1.zip › Case 1/Rectum MLH1.jpeg]

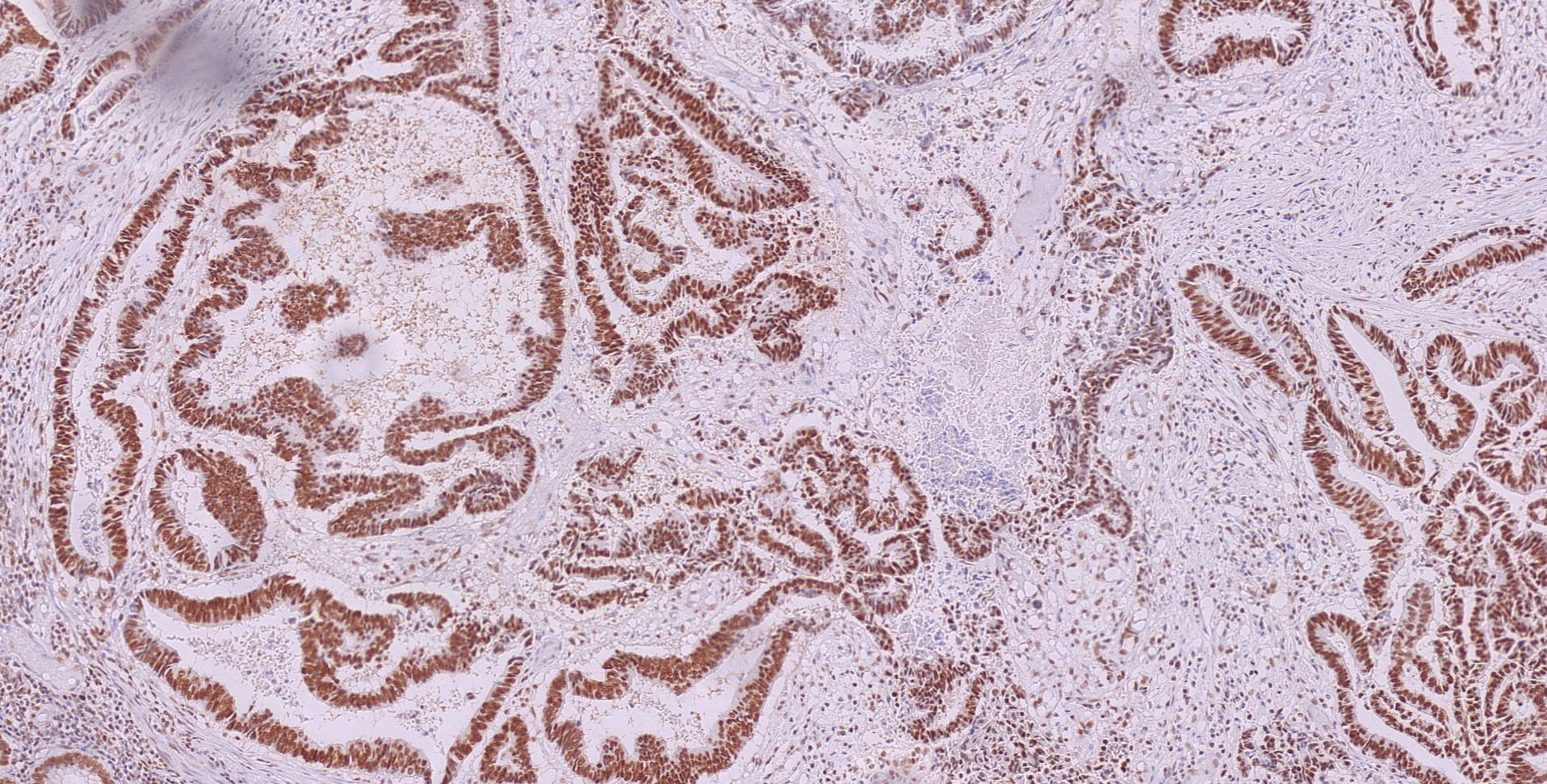

Supplement: Supplementary file 1 [file DataSheet_1.zip › Case 1/Rectum MSH2.jpeg]

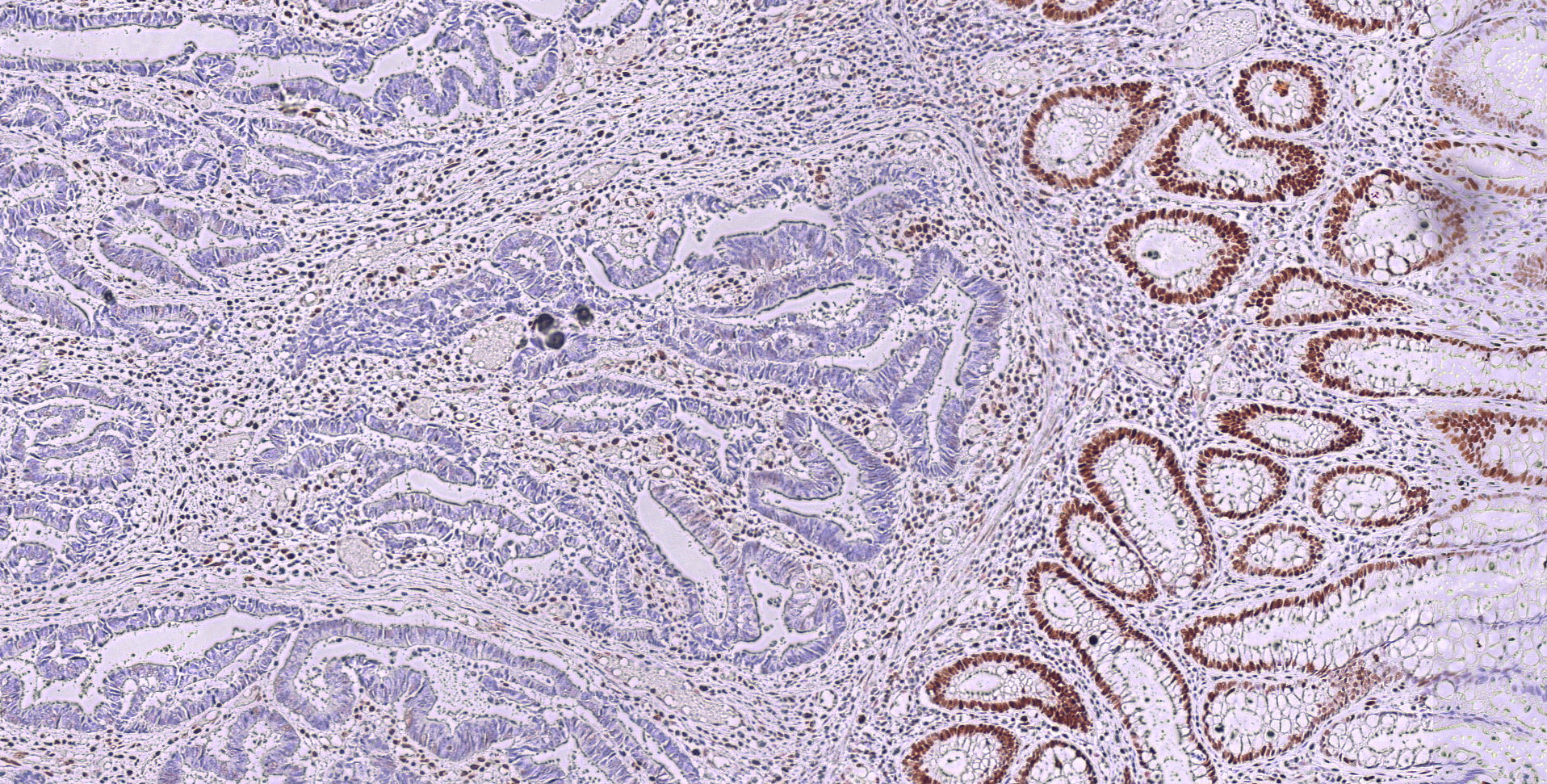

Supplement: Supplementary file 1 [file DataSheet_1.zip › Case 1/Rectum MSH6.jpeg]

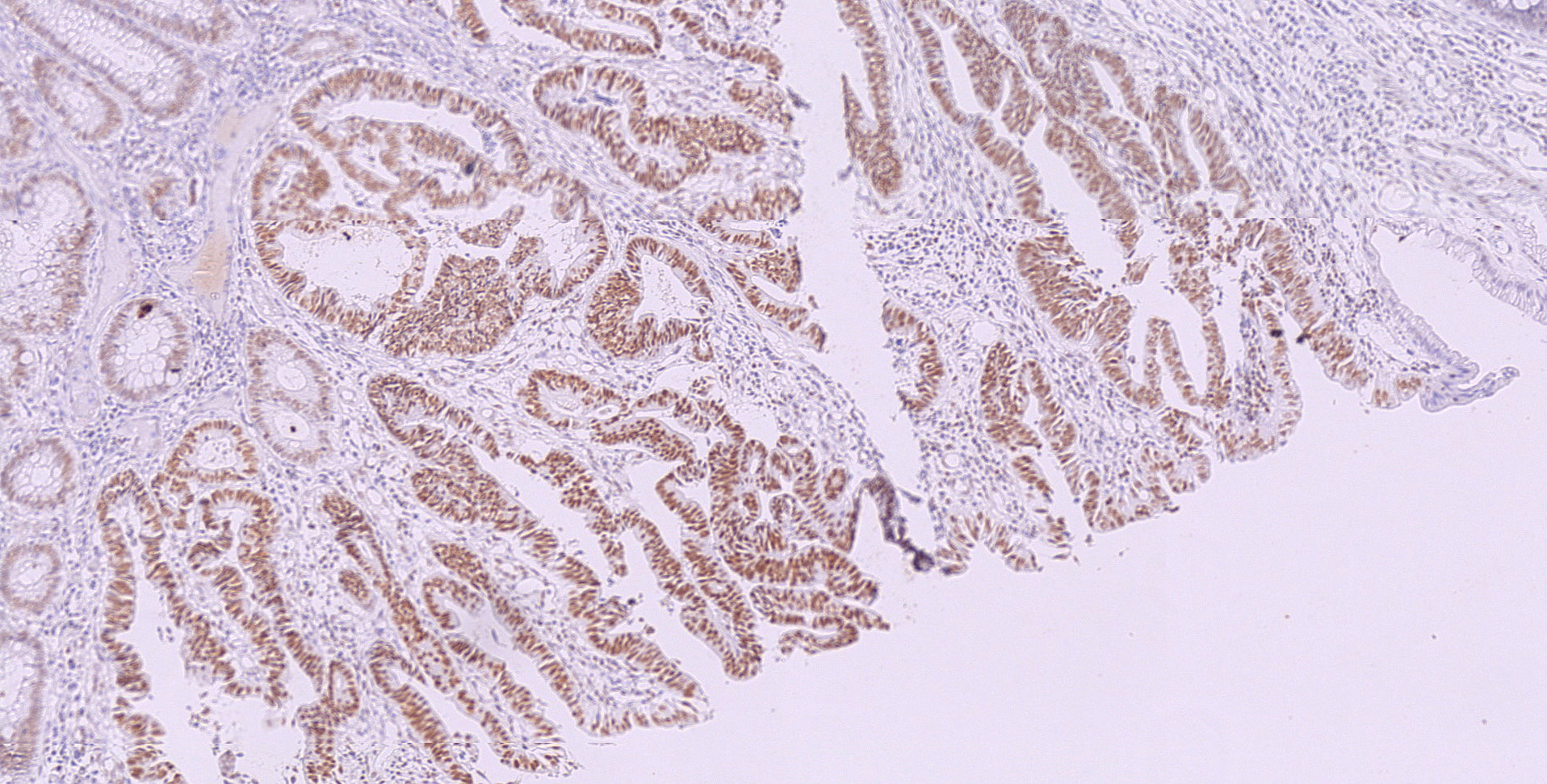

Supplement: Supplementary file 1 [file DataSheet_1.zip › Case 1/Rectum PMS2.jpeg]

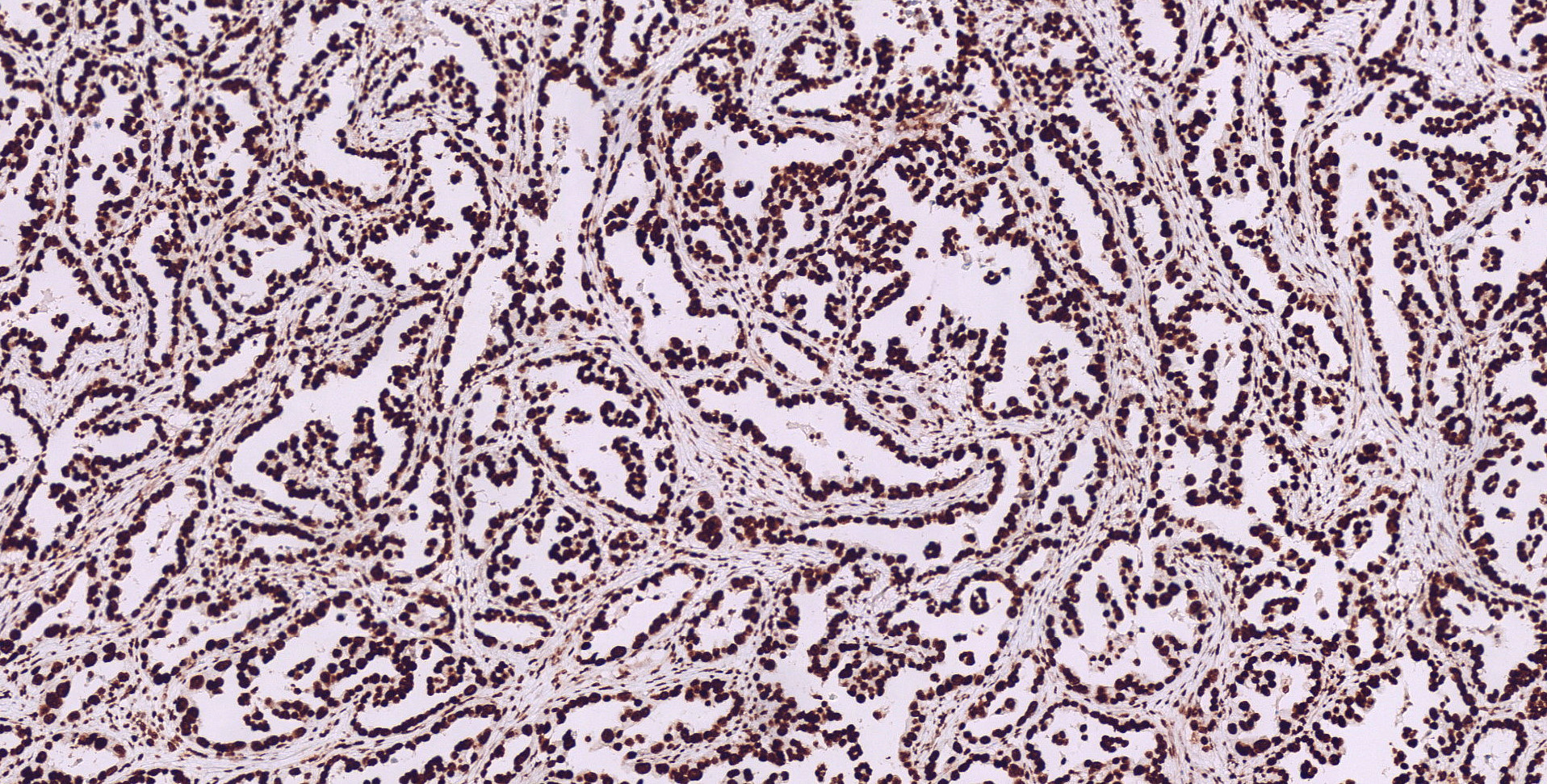

Supplement: Supplementary file 1 [file DataSheet_1.zip › Case 1/Right lower abdominal mass MLH1.jpeg]

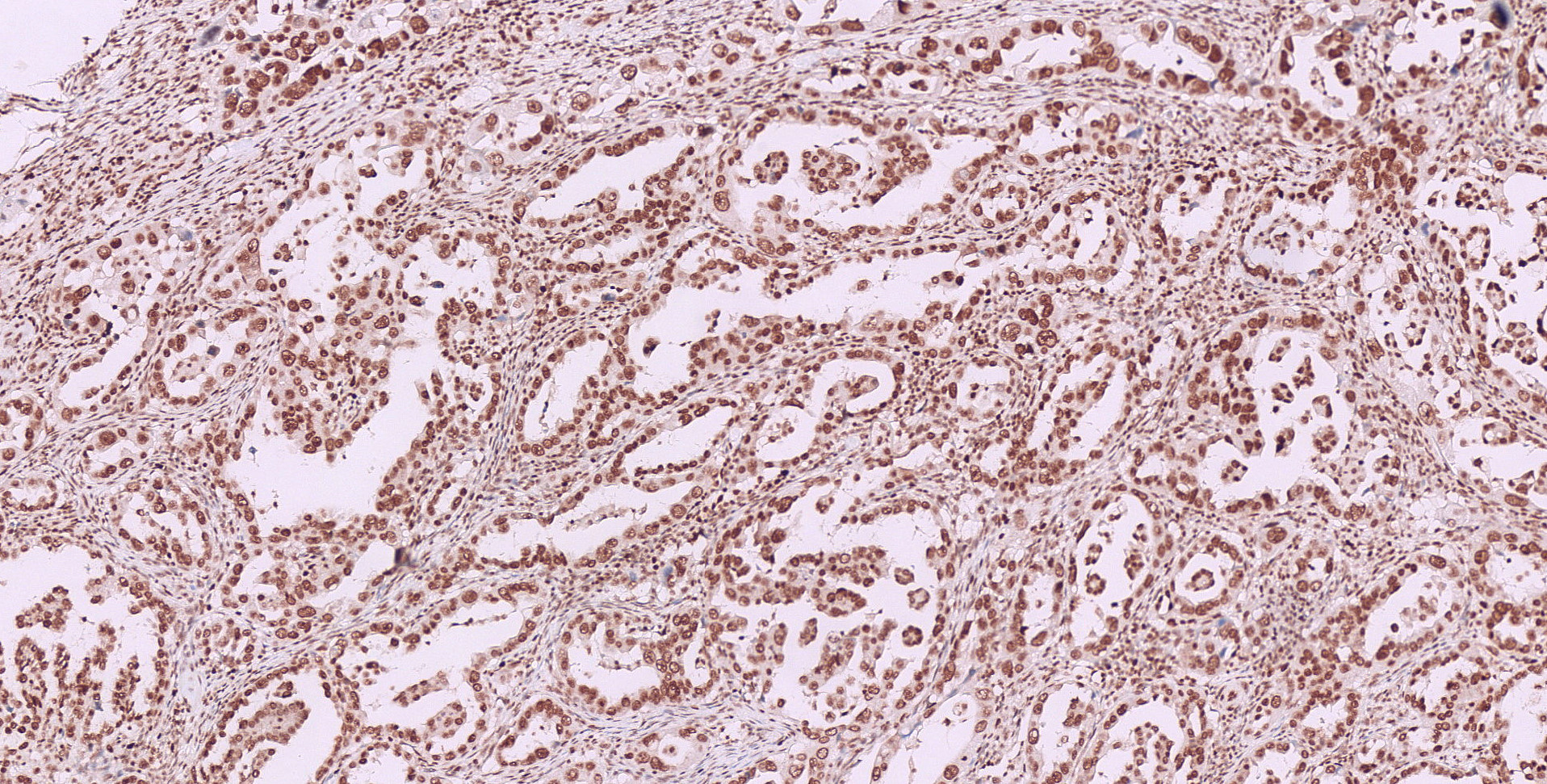

Supplement: Supplementary file 1 [file DataSheet_1.zip › Case 1/Right lower abdominal mass MSH2.jpeg]

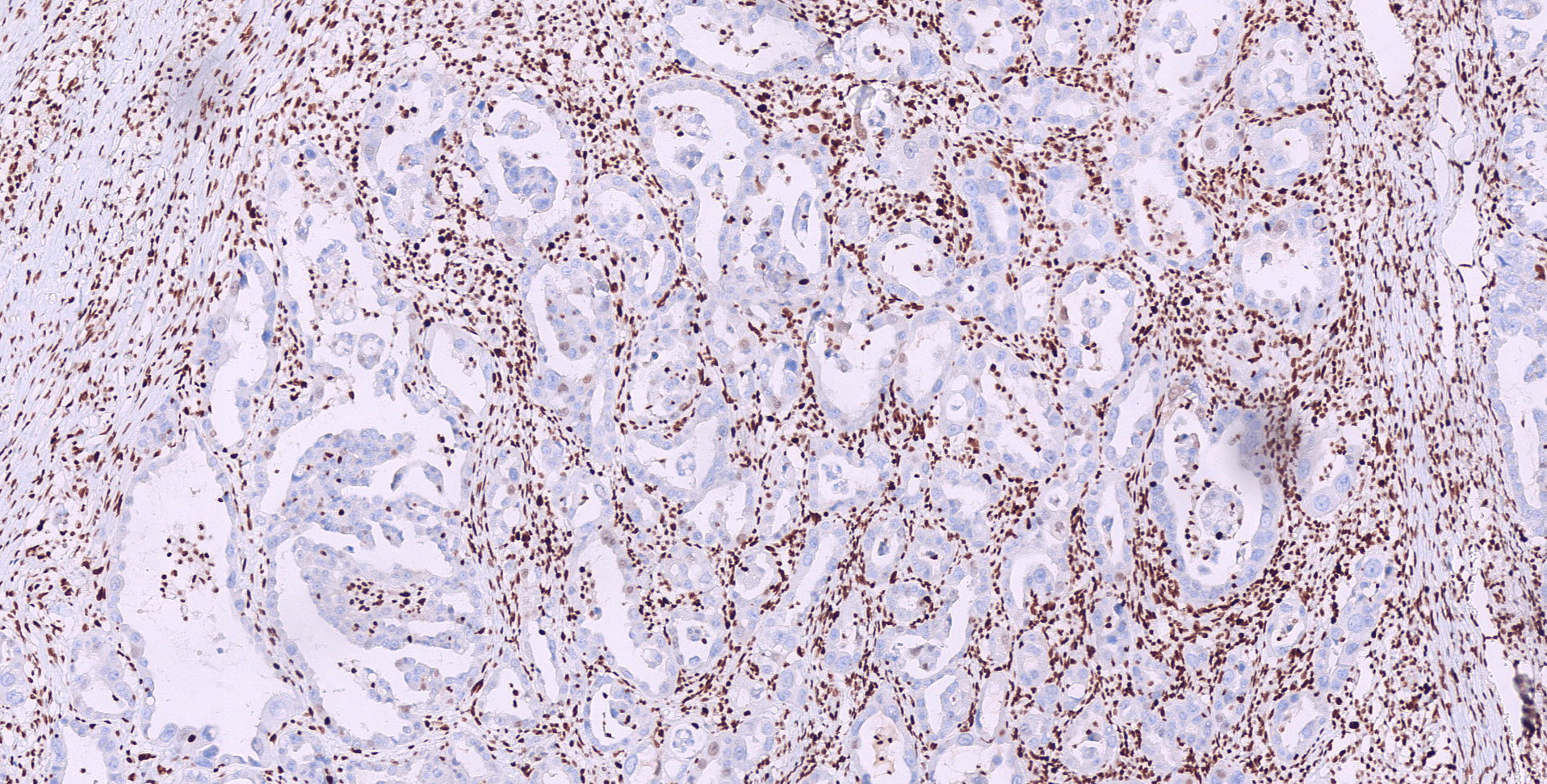

Supplement: Supplementary file 1 [file DataSheet_1.zip › Case 1/Right lower abdominal mass MSH6.jpeg]

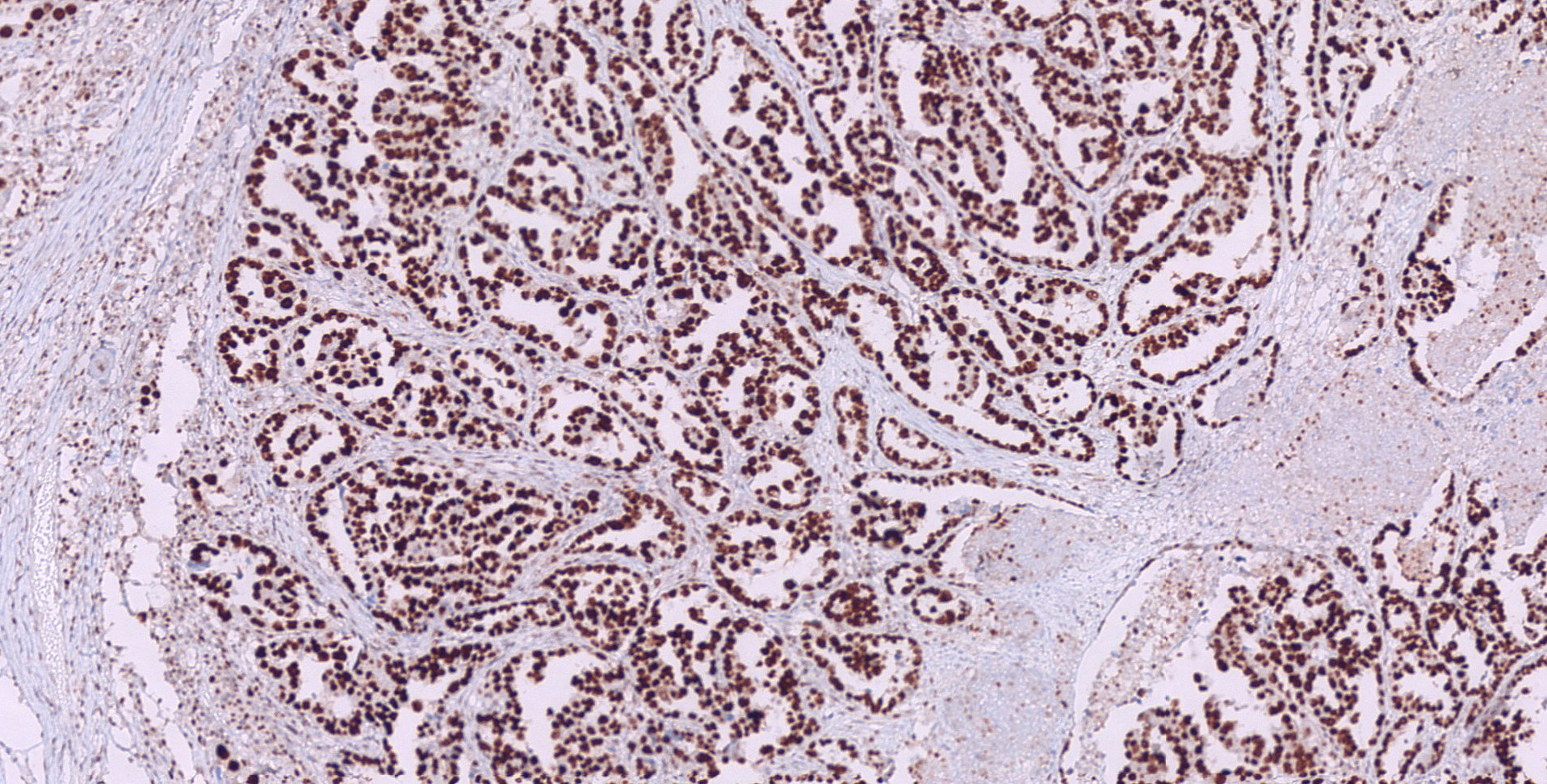

Supplement: Supplementary file 1 [file DataSheet_1.zip › Case 1/Right lower abdominal mass PMS2.jpeg]
